# Mechanisms of acquired resistance to EGFR-tyrosine kinase inhibitor in Korean patients with lung cancer

**DOI:** 10.1186/1471-2407-13-606

**Published:** 2013-12-27

**Authors:** Wonjun Ji, Chang-Min Choi, Jin Kyung Rho, Se Jin Jang, Young Soo Park, Sung-Min Chun, Woo Sung Kim, Jung-Shin Lee, Sang-We Kim, Dae Ho Lee, Jae Cheol Lee

**Affiliations:** 1 Department of Pulmonary and Critical Care Medicine, Asan Medical Center, University of Ulsan College of Medicine, Seoul, Korea; 2 Department of Oncology, Asan Medical Center, University of Ulsan College of Medicine, 88, Olympic-ro 43-gil, Seoul, Songpa-gu, Korea; 3 Department of Pathology, Asan Medical Center, College of Medicine, University of Ulsan, Seoul, Korea

**Keywords:** Non-small cell lung carcinoma, Epidermal growth factor receptor mutation, EGFR tyrosine kinase inhibitor, Acquired resistance, Resistant mechanism, Mass spectrometric genotyping

## Abstract

**Background:**

Despite an initial good response to epidermal growth factor receptor (EGFR)-tyrosine kinase inhibitor (TKI), resistance to treatment eventually develops. Although several resistance mechanisms have been discovered, little data exist regarding Asian patient populations.

**Methods:**

Among patients at a tertiary referral hospital in Korea who initially responded well to gefitinib and later acquired resistance to treatment, we selected those with enough tissues obtained before EGFR-TKI treatment and after the onset of resistance to examine mutations by mass spectrometric genotyping technology (Asan-Panel), *MET* amplification by fluorescence in situ hybridization (FISH), and analysis of AXL status, epithelial-to-mesenchymal transition (EMT) and neuroendocrine markers by immunohistochemistry.

**Results:**

Twenty-six patients were enrolled, all of whom were diagnosed with adenocarcinoma with *EGFR* mutations (19del: 16, L858R: 10) except one (squamous cell carcinoma with 19del). Secondary T790M mutation was detected in 11 subjects (42.3%) and four of these patients had other co-existing resistance mechanisms; increased AXL expression was observed in 5/26 patients (19.2%), *MET* gene amplification was noted in 3/26 (11.5%), and one patient acquired a mutation in the phosphatidylinositol-4, 5-bisphosphate 3-kinase catalytic subunit alpha isoform (*PIK3CA*) gene. None of the patients exhibited EMT; however, increased CD56 expression suggesting neuroendocrine differentiation was observed in two patients. Interestingly, conversion from L858R-mutant to wild-type *EGFR* occurred in one patient. Seven patients (26.9%) did not exhibit any known resistance mechanisms. Patients with a T790M mutation showed a more favorable prognosis.

**Conclusion:**

The mechanisms and frequency of acquired EGFR-TKI resistance in Koreans are comparable to those observed in Western populations; however, more data regarding the mechanisms that drive EGFR-TKI resistance are necessary.

## Background

Lung cancer is the leading cause of cancer deaths [1]. Three out of four patients present with advanced-stage disease, and the prognosis is generally poor. However, recent advances with targeted therapies, such as epidermal growth factor receptor (EGFR)-tyrosine kinase inhibitors (TKIs), have resulted in marked benefit to subsets of lung cancer patients whose tumors have specific genetic mutations. However, despite the initial beneficial effect of EGFR-TKI treatment, most patients with non-small cell lung cancer (NSCLC) eventually develop resistance to EGFR-TKIs, with a median time to disease progression of about 12 months [2,3]. Secondary biopsy of growing tumors at the onset of clinical progression is crucial for identifying the mechanisms of resistance, although this is often not easily accomplished.

Recent efforts to develop strategies for overcoming acquired resistance to EGFR-TKIs have identified severalresistance mechanisms. Approximately half of the cases of acquired resistance are mediated by a secondary T790M mutation on exon 20 of the *EGFR* gene [4-6]. In addition, amplification of the *MET* gene has been reported to contribute to resistance in approximately 5–20% of cases [6-8] and increased AXL expression was recently discovered to occur in almost 20% of patients [9] phosphatidylinositol-4, 5-bisphosphate 3-kinase catalytic subunit alpha isoform (*PIK3CA*) mutation, epithelial-to-mesenchymal transition (EMT) and small cell lung cancer (SCLC) transformation are also associated with acquired resistance [6]. Although some studies have examined the mechanisms and frequency of EGFR-TKI resistance, little data exists regarding Asian populations of cancer patients. The aim of this study was to analyze the mechanisms of acquired resistance to EGFR-TKI and its frequency in Korean patients with lung cancer.

## Methods

### Patients

We reviewed the medical records of patients with NSCLC with *EGFR* mutations and acquired resistance to EGFR-TKI between 2007 and 2010. All patients fulfilled the definition of acquired resistance to EGFR-TKI [10], which was defined as having received treatment with a single agent EGFR-TKI, exhibiting objective clinical benefit from treatment, and then experiencing disease progression while under continuous treatment with EGFR-TKI. At the time drug resistance developed, some patients underwent post-resistance biopsy for evaluation of the mechanisms of resistance. We selected patients from whom the tissues obtained both before EGFR-TKI treatment and after resistance were sufficient to assess *EGFR, KRAS, BRAF,* and *PIK3CA* mutations by “Asan-Panel” analysis, perform fluorescence in situ hybridization (FISH) to identify *MET* amplification, and examine AXL status, EMT and neuroendocrine markers by immunohistochemistry. All patients provided informed consent, and the study was approved by the Institutional Review Board of the Asan Medical Center (Approval Number: 2011–0526).

### Mutation analysis

A mass spectrometric genotyping technology, called the “Asan-Panel”, was used for genetic analysis. First, DNA was extracted from paraffin-embedded tissues using QIAamp DNA FFPE tissue kit (#56404; Qiagen, Hilden, Germany) according to the manufacturer’s protocol. DNA quantity was measured using the Quant-iT™ PicoGreen® dsDNA Assay kit (Invitrogen, Carlsbad, CA) andbrought to a final concentration of 5 ng/μl. Mutation analysis using the Asan-Panel was performed under the SequenomMassARRAY technology platform with iPLEX-Pro chemistry (Sequenom, San Diego, USA). The protocols that were previously performed as “OncoMap” [11-13] were followed with minor modifications. In brief, specific assay pools were designed using AssayDesignersoftware in MassARRAY Typerpackage software (v4.0) with filters for proximal single nucleotide polymorphisms (SNPs) and assessment of the specificity of PCR amplification and the subsequent primer extension reaction. To decrease the number of multiplex PCR tubes, manual modification of some PCR primers and extension probes was conducted. A total of 59 amplicons were amplified in eight different multiplex pools with an average of 8-plex. After multiplex PCR, residual deoxynucleotides were inactivated by incubation with Shrimp Alkaline Phosphatase (Catalog No. 10142–2, Sequenom). Single-base extension (SBE) reaction products using a mixture of mutation site-specific probes were then spotted onto a 384-format SpectroCHIP II with the MassARRAY Nanodispenser. Mass determination was performed with the MassARRAY Analyzer Compact MALDI-TOF mass spectrometer, and MassARRAY Typer 4.0 software was used for data acquisition and analysis. Genotypes were called after cluster analysis using the default setting of the Gaussian mixture model. Genotype calls were then reviewed manually to identify any uncertain calls due to clustering artifacts. A total of 87 genetic mutations located in *EGFR, KRAS, BRAF* and *PIK3CA* genes were examined by Asan-Panel analysis.

### FISH analysis for *MET* amplification

For FISH, 2 μm-thick sections from each paraffin block were prepared. Deparaffinization, pre-treatment and protease digestion procedures were performed following the Abbott Vysis D7S522/CEP 7 FISH probe kit protocol (Abbott Laboratories, Abbott Park, Des Plaines, IL, USA). Probe mixtures were hybridized at 37°C for 14 to 18 hours. After hybridization, slides were washed in 2× SSC/0.3% NP-40 at 72°C for 2 min, air dried, and counterstained with 4,6-diamidino-2-phenylindole (DAPI). The slides were examined under a fluorescence microscope (Olympus, Tokyo, Japan) equipped with Spectrum Orange/Green dual and DAPI single filters. The slides were stored at -20°C until examination. A *c-met/CEP7* ratio was established on the basis of a count of at least 60 cells by enumerating both orange (*c-met*) and green (chromosome 7, *CEP7*) signals. Samples with a *c-met/CEP7* ratio greater than 2 were considered to have *MET* amplification.

### Immunohistochemistry for AXL, EMT and neuroendocrine markers

All biopsy specimens underwent histologic review after H&E and immunohistochemical staining for specific markers, such as thyroid transcription factor 1 (TTF-1). For immunohistochemical analysis, paraffin sections (4 μm thick) were deparaffinized with xylene, rinsed thoroughly with ethanol, and then soaked in 0.03% hydrogen peroxide in methanol to inactivate the endogenous peroxidase activity. The sections were incubated with either 10% goat serum or 10% rabbit serum, and then incubated with the primary antibodies. The sections were washed with phosphate-buffered saline (PBS) and processed using a DAKO EnVision kit (DAKO, Los Angeles, CA), as directed by the manufacturer. The color was developed with 3,3′-diaminobenzindine (DAB) containing 0.3% H_2_O_2_. Primary antibodies against the following antigens were used: CD56, synaptophysin and chromogranin (Santa Cruz Biotechnology, Santa Cruz, CA) for SCLC transformation; E-cadherin and vimentin (Calbiochem, San Diego, CA) for EMT; AXL and p-AXL (R&D Systems, Minneapolis, MN) for AXL status.

## Results

### Baseline clinical and molecular characteristics

Twenty-six patients were eligible for this study; of these, 10 patients (38.5%) were male and 16 (61.5%) were female. The median age was 58-years-old. All patients except one were diagnosed with adenocarcinoma of the lung with *EGFR* mutation at initial diagnosis. One patient had squamous cell carcinoma with a deletion mutation on exon 19 of *EGFR*. The deletion mutation on exon 19 of *EGFR* gene was present in 16 patients (61.5%), while the L858R point mutation on exon 21 was noted in 10 (38.5%). All patients were treated with gefitinib and showed a partial response. The secondary biopsy sites were lung (65.4%), mediastinal or cervical lymph nodes (19.2%), liver (7.7%), malignant pleural effusion (3.8%), and bone (3.8%). The biopsy site after resistance was same as the initial site in 15 patients (Table 1).

**Table 1:** Baseline characteristics, clinical course and mechanism of acquired resistance to EGFR-TKI in 26 patients

**SN**	**Sex**	**Age**	**Histology**	***EGFR mutation***	**Changes in 2**^**nd **^**Bx**	**TKI**	**PFS (months)**	**OS (months)**	**Bx site**	
									**1st**	**2nd**
1	M	80	ACC	19 del	T790M + H1047L (PIK3CA)	Gefitinib	34.7	65.7	RUL	RUL
2	F	40	ACC	19 del	T790M	Gefitinib	12.7	17.1	LLL	LN
3	F	48	ACC	L858R	T790M + AXL	Gefitinib	11.5	38.9	LLL	RLL
4	F	64	SqCC	19 del	T790M + MET amp	Gefitinib	7.8	14.2	RUL	RUL
5	F	73	ACC	L858R	T790M + MET amp	Gefitinib	13.1	47.7	RLL	RLL
6	M	67	ACC	L858R	T790M	Gefitinib	16.7	29.1	RUL	Liver
7	M	53	ACC	19 del	T790M	Gefitinib	15.8	79.4	LUL	LUL
8	F	48	ACC	19 del	T790M	Gefitinib	18.9	50.9	LN	LN
9	F	57	ACC	19 del	T790M	Gefitinib	16.2	37.2	LLL	Effusion
10	M	56	ACC	19 del	T790M	Gefitinib	14.8	28.9	RUL	RUL
11	F	54	ACC	19 del	T790M	Gefitinib	28.7	58.2	RML	LN
12	M	49	ACC	L858R	WT EGFR	Gefitinib	6.6	89.2	RUL	Liver
13	F	50	ACC	19 del	MET amp	Gefitinib	10.7	35.2	LN	LUL
14	F	64	ACC	L858R	AXL	Gefitinib	25.4	94.6	LUL	RUL
15	M	67	ACC	19 del	AXL	Gefitinib	11.4	30.2	LUL	Bone
16	M	59	ACC	19 del	AXL	Gefitinib	4.3	41.3	RLL	LN
17	F	76	ACC	19 del	AXL	Gefitinib	10.7	31.0	RUL	RUL
18	F	72	ACC	19 del	None	Gefitinib	26.1	46.4	RML	RML
19	F	51	ACC	L858R	None	Gefitinib	6.1	23.0	Bone	LN
20	M	63	ACC	L858R	None	Gefitinib	5.0	9.6	RLL	RLL
21	F	73	ACC	L858R	None	Gefitinib	3.9	25.1	LN	LN
22	F	66	ACC	L858R	None	Gefitinib	4.8	21.2	LUL	LUL
23	F	57	ACC	19 del	None	Gefitinib	11.0	36.2	LUL	LUL
24	M	65	ACC	L858R	None	Gefitinib	5.4	20.6	RLL	RLL
25	F	56	ACC	19 del	CD56↑	Gefitinib	8.7	32.3	RUL	RUL
26	M	50	ACC	19 del	CD56↑	Gefitinib	5.9	15.4	LUL	LUL

*ACC*, Adenocarcinoma; *SqCC*, Squamous cell carcinoma; *TKI*, Tyrosine kinase inhibitor; *PFS*, Progression-free survival; *OS*, Overall survival; *Bx*, Biopsy; *RUL*, Right upper lobe; *RML*, Right middle lobe; *RLL*, Right lower lobe; *LUL*, Left upper lobe; *LLL*, Left lower lobe; *LN*, Lymph node; *WT*, Wild-type; ↑, increased expression.

### Resistance mechanisms to EGFR-TKI

**Figure 1:**
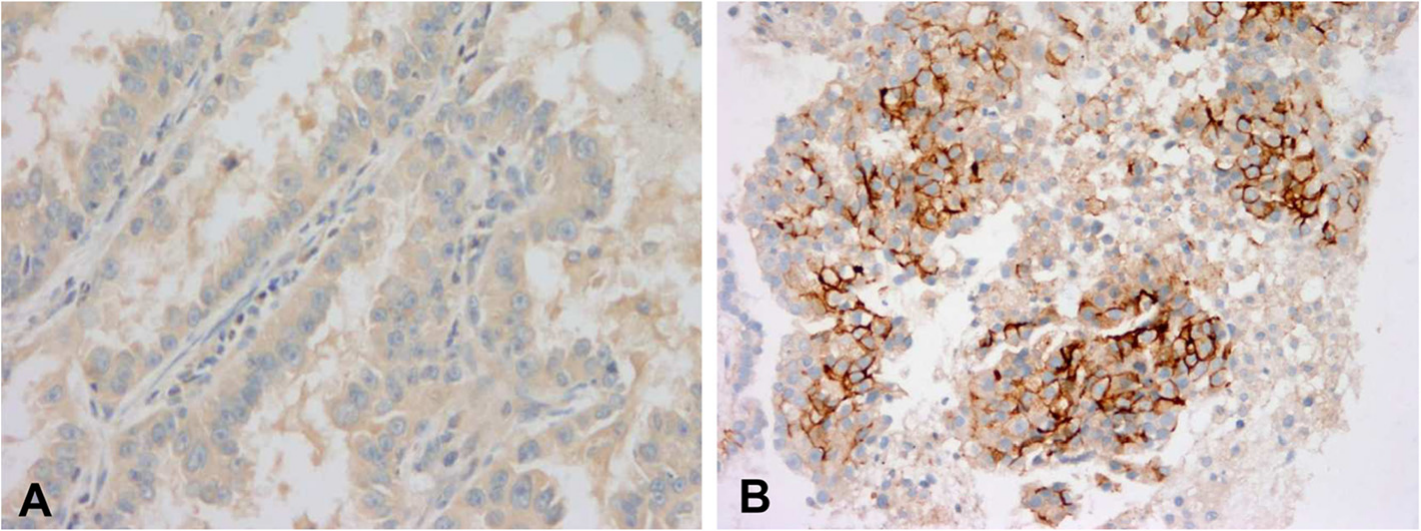
**Representative photomicrographs of AXL immunohistochemical staining. (A)** The original tumor cells before EGFR-TKI treatment were negative for AXL in immunohistochemical staining. **(B)** Tumor cells resistant to EGFR-TKI were immuno-positive for AXL.

**Figure 2:**
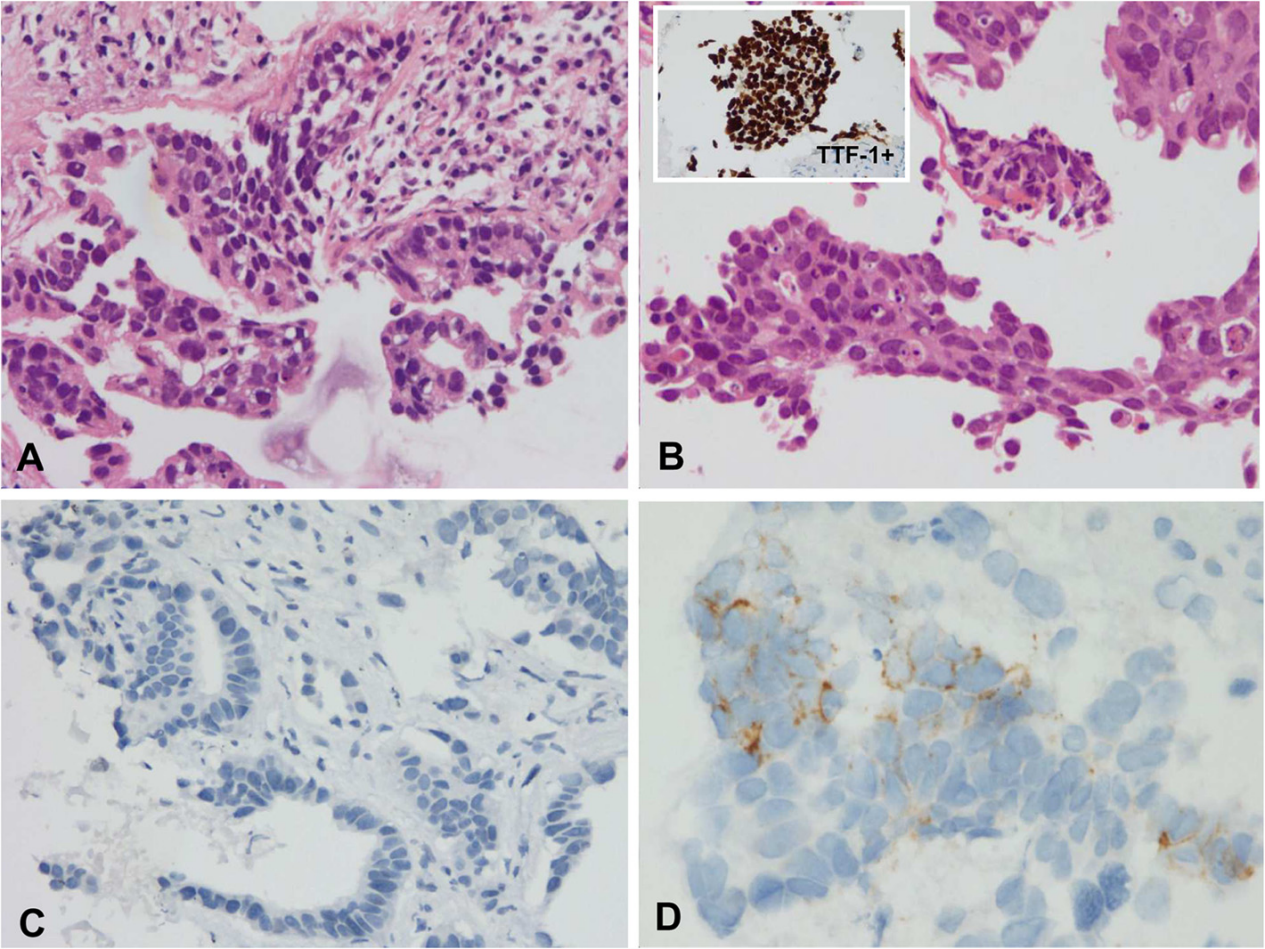
**Increased CD56 expression in two patients. (A)** The initial biopsy showed typical adenocarcinoma in H&E staining. **(B)** There were no significant morphological changes of tumor cells with persistent immuno-positivity for TTF-1 after resistance. **(C)** The expression of CD56 was negative in initial sample. **(D)** CD56 expression in some cells of tissues obtained after resistance was increased.

**Figure 3:**
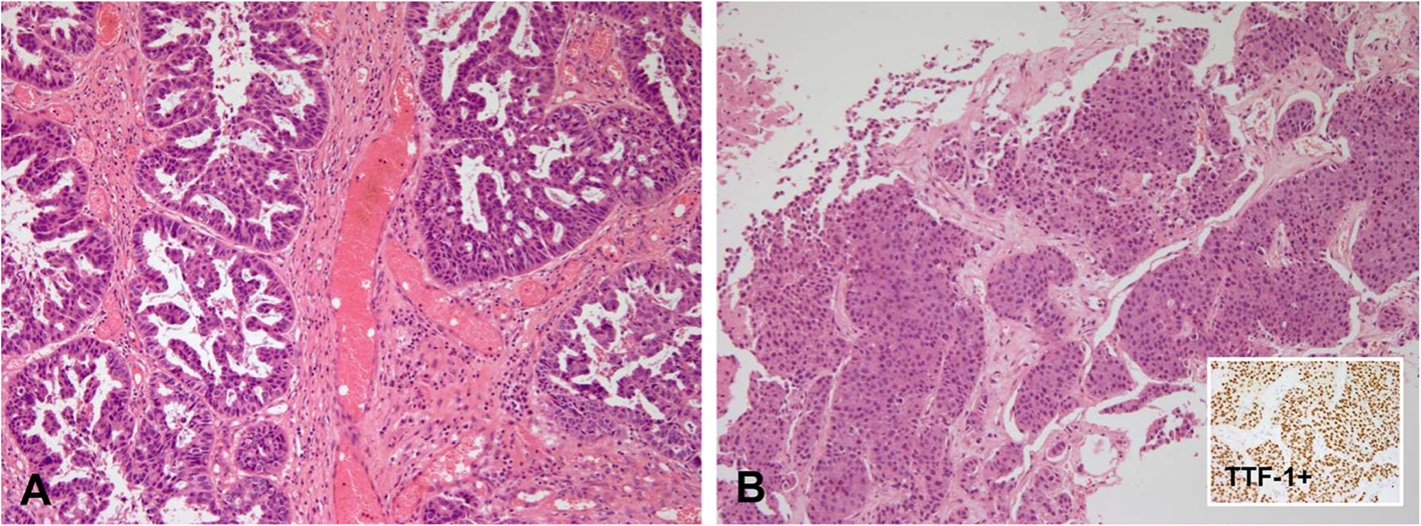
**Histomorphological changesin tumor cells after conversion to wild-type *EGFR*. (A)** Tumor cells formed a glandular configuration when they harbored the L858R *EGFR* mutation. **(B)** Tumor cells were clustered in a compact solid pattern after they converted to wild-type *EGFR*-expressing cells. These tumor cells strongly expressed TTF-1, confirming that it is still adenocarcinoma.

**Figure 4:**
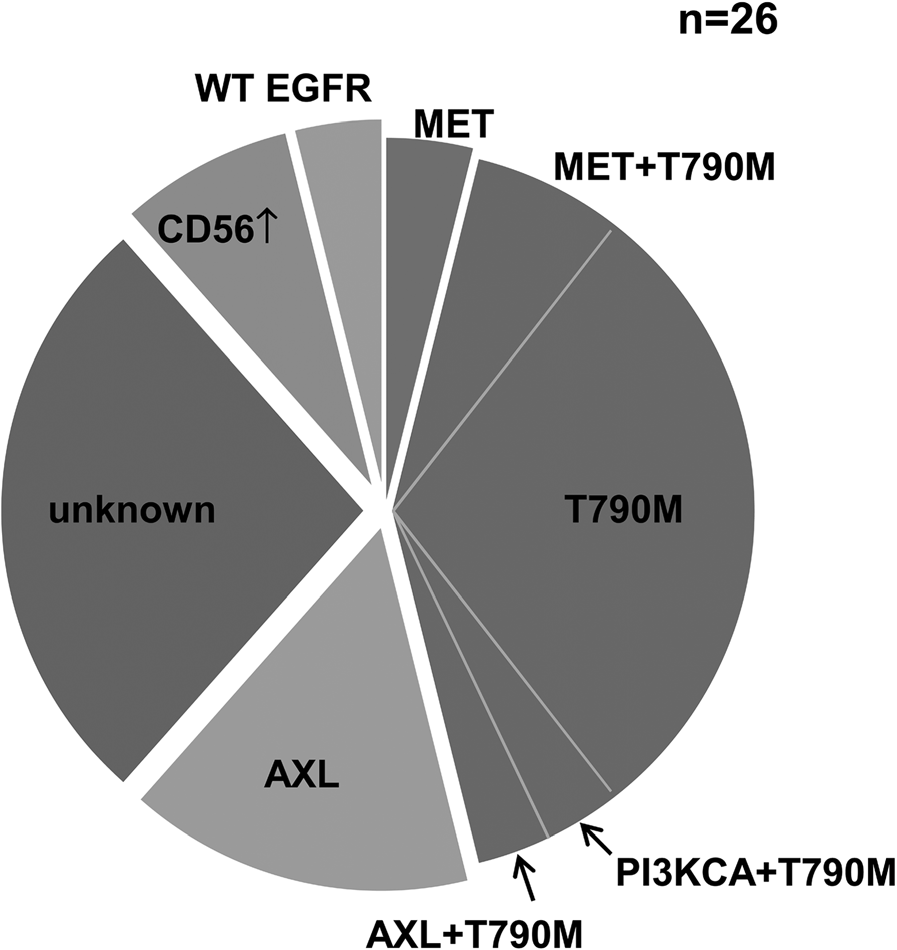
**The frequency of acquired EGFR-TKI resistance in 26 patients.** Secondary T790M mutation was the most common mechanism, found in 11 patients (42.3%). Four patients had other co-existing resistant mechanisms (*MET*:2, *AXL*:1, *PI3KCA*:1). Increased *AXL* expression was observed in 5/26 patients (19.2%), while *MET* gene amplification was noted in 3/26 patients (11.5%). One patient acquired a mutation in the *PIK3CA* gene and 2 patients showed increased CD56 expression, suggesting neuroendocrine differentiation. Conversion from L858R-mutant to wild-type *EGFR*-expressing cells occurred in 1 patient, and 7 patients (26.9%) did not exhibit any known resistance mechanisms.

Secondary T790M mutation was detected in 11 patients (42.3%), four of which had additional resistance mechanisms: *MET* amplification was observed in two patients, increased AXL expression in one patient, and *PIK3CA* mutation in one patient. Increased AXL expression (Figure 1) was seen in 5/26 patients (19.2%), while *MET* gene amplification was noted in 3/26 patients (11.5%). One patient acquired an H1047L point mutation in the *PIK3CA* gene, which was accompanied by the T790M mutation. No patient exhibited evidence of EMT, whereas increased CD56 expression suggesting neuroendocrine differentiationwas observed in two patients. However, the morphologic change and expression of synaptophysin and chromogranin was not evident in these patients (Figure 2). Interestingly, conversion from L858R-mutant to wild-type *EGFR* occurred in one patient (Figure 3). Seven of the patients (26.9%) did not exhibit any known EGFR-TKI resistance mechanisms. The frequency of resistance mechanisms is shown in Figure 4.

### Outcomes

**Figure 5:**
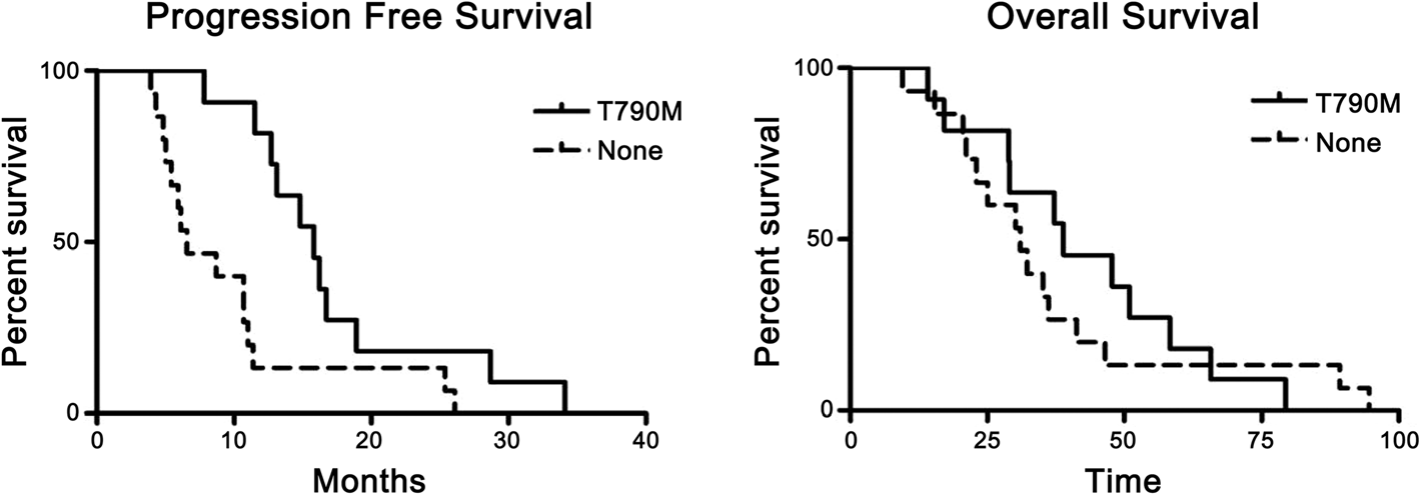
**Progression-free survival (PFS) and overall survival (OS) according to the T790M mutation.** PFS was significantly better in patients with secondary T790M mutation than in those without T790M (15.8 months vs 6.6 months, p = 0.009), while OS was not statistically different (38.9 months vs 38.9 months, p = 0.617).

Median progression-free survival (PFS) following gefitinib treatment was 11 months, and the median overall survival (OS) time was 32.3 months. PFS was significantly better in patients with secondary T790M mutation than in those without T790M (p = 0.009, Figure 5), while OS was not statistically different (p = 0.617, Figure 5).

## Discussion

In this study, we explored themechanisms of resistance to EGFR-TKI and their frequency in a Korean population. Because biopsy after disease progression following EGFR-TKI treatment is often challenging, few studies regarding the onset of EGFR-TKI resistance exist, and this is especially true of EGFR-TKI resistance in Asian populations, even though *EGFR* mutations in Asian patients are frequent.

Similar to the data published in previous reports [6,14], we observed that secondary T790M mutation was the most common mechanism of EGFR-TKI resistance, representing 43.9% of all cases. The sensitivity of mass spectrometric genotyping technologies such as OncoMap or Asan-Panel is known to be approximately 1–5% [6,15], and so detection of the T790M mutation could be increased if more sensitive techniqueswere used. Interestingly, four patients with T790M had co-existing resistance mechanisms such as *MET* amplification, increased AXL expression and *PIK3CA* mutation. Simultaneous occurrence of two resistant mechanisms has been reported by several investigators. For example, Sequist LV et al. showed that some patients with a T790M mutation exhibited other possible contributing factors to resistance, such as *EGFR* amplification or *β-catenin* and *APC* mutation [6]. In addition, among 10 EGFR-TKI-resistant tumors from nine patients with *MET* amplification, four also expressed *EGFR* with the T790M mutation [8]. Supporting this, the H820 cell line harboring both sensitizing (19del) and resistant (T790M) *EGFR* mutations also contains *MET* amplification and is more sensitive to MET inhibition than EGFR inhibition [8]. Secondary T790M mutation seems to be acquired by the selection of pre-existing populations of T790M-harboring cells among the total tumor cells during the period of treatment with EGFR-TKI [16,17]. During this time, tumor cells possessing a resistance mechanism in addition to T790M could be more favorably selected.

Oxnard GR et al. reported that patients with tumors harboring T790M mutation had more favorable outcomes, and that those without T790M more frequently progress to previously uninvolved organs. They suggested that the favorable outcome could be related tothe indolent growth of tumors containing the T790M mutation [18]. In agreement with these findings, we also observed that patients with secondary T790M mutation showed significantly longer progression-free survival (*p = 0.009*).

Recently, we demonstrated that increased AXL expression could contribute to erlotinib-resistance in both cell lines and an animal model. Altered AXL-related signaling was also observed in approximately 20% of patients with acquired resistance to EGFR-TKI, although it remains to be determined whether these patients could benefit from AXL inhibition [9]. In EGFR-TKI resistance, AXL could act as a bypass to activate downstream signals related to cell survival and growth. Therefore, combined treatment with EGFR and AXL inhibitors might effectively abrogate the growth of tumor cells. A similar phenomenon can be observed in MET-mediated resistance, as shown in a previous report by Engelman JA et al. [7]. Although the frequency of *MET* amplification in cases of EGFR-TKI resistance was initially reported to be 20% [7], this has varied by approximately 5–11% in follow-up studies [6,14,19]. Similarly, the exact frequency of AXL-mediated resistance should be determined by further investigation.

Sequist LV et al. found that 14% of biopsy specimens taken at the onset of resistance showed morphologies similar to SCLC, as well as increased expression of neuroendocrine markers such as CD56, synaptophysin and chromogranin. In their study, three patients treated with conventional chemotherapeutic agents for SCLC, including etoposide and cisplatin, responded well [6]. In another study, biopsy after the onset of resistance showed that approximately 3% of NSCLC tumors exhibited morphological transformation to small cell or high grade neuroendocrine carcinomas [14]. These findings suggest that transformation to SCLC or neuroendocrine carcinoma could be a possible mechanism of resistance. Although pulmonary alveolar cells have been found to transform occasionally to a small cell morphology when loss of *p53* and *Rb1* is induced [20], the biological underpinning of the SCLC transformation is unknown. In our study, we observed increased CD56 expression in 7.7% of patients. However, because it was not accompanied by the morphologic change and upregulation of other neuroendocrinemarkers, such as synaptophysin and chromogranin, the reason for this is not clear.

Other possible resistance mechanisms, specifically *PIK3CA* mutation and conversion to wild-type *EGFR* were noted in some cases, although *PIK3CA* mutation concomitantly occurred with T790M mutation. In a previous *in vitro* study, gefitinib-induced apoptosis was abrogated when *PIK3CA* mutation was introduced in HCC827 cells with a deletion mutation in exon 19 of the *EGFR* gene [21]. Moreover, Sequist LV et al. reported cases of EGFR-TKI resistance in tumors with a *PIK3CA* mutation [6]. Thus, although *PIK3CA* mutation may be a contributing factor to EGFR-TKI resistance, it is not frequent. Some studies have reportedthe loss of EGFR-activating gene mutations in resistant tumor samples [22,23]. This could happen through the selection of pre-existing tumor cells expressing wild-type *EGFR* during EGFR-TKI treatment, similar to the effect of the T790M mutation. However, because *EGFR* mutation is considered to be a driver mutation for carcinogenesis, the presence of another driving factor to induce tumor cells with wild-type *EGFR* would be necessary, suggesting that this event would be very rare.

As the data about resistant mechanisms have been accumulated, the procurement of resistant samples to guide following treatments is becoming more important. However, the performing the re-biopsy is not so easy in clinical practice. Attempts to use circulating tumor cells or circulating free DNAs in bloods or other body fluids (“so-called liquid biopsy”) are currently in progress because those are non-invasive, convenient and can be performed repeatedly [24,25]. Technical advances in tests and processing samples would help this liquid biopsy to have broad clinical applications, especially in elucidation of resistant mechanisms.

## Conclusions

Our study is the first to present data regarding EGFR-TKI resistance mechanisms and their frequency in a Korean population. The mechanisms and frequency of acquired EGFR-TKI resistance in Koreans are comparable to those observed in Western populations. However, this study is limited by the small number of patients. In addition, it is a single center study that utilized retrospective analysis, making generalization of the results difficult. Therefore, a greater effort to procure appropriate tissues in instances of acquired EGFR-TKI resistance will be necessary for the results presented here to be confirmed by more extensive studies. Although secondary biopsy is difficult at the time of disease progression and the exact timing for secondary biopsy should be determined, these efforts will provide the data necessary to develop strategies for overcoming EGFR-TKI resistance, leading to a better prognosis for patients with lung cancer.

## Abbreviations

EGFR: Epidermal growth factor receptor; TKI: Tyrosine kinase inhibitor; FISH: Fluorescence in situ hybridization; PI3KCA: Phosphatidylinositol-4, 5-bisphosphate 3-kinase catalytic subunit alpha isoform; EMT: Epithelial-to-mesenchymal transition; NSCLC: Non-small cell lung cancer; SCLC: Small cell lung cancer; TTF-1: Thyroid transcription factor 1; PFS: Progression-free survival; OS: Overall survival.

## Competing interests

The authors have no financial/non-financial competing interest with any companies/organizations whose products or services may be discussed in this article.

## Authors’ contributions

WJJ and JCL had full access to the data and take full responsibility for the content of this manuscript. CMC contributed to the study design, obtained biopsy tissue specimens from patients, and participated in the interpretation of results and drafting of the manuscript. JKR contributed to the study design, interpretation of the results and drafting of the manuscript. SJJ and YSP contributed to the review of pathologic findings, FISH analysis of MET, immunohistochemical analysis of AXL, interpretation of the results and drafting of the manuscript. SMC contributed to mutation analysis using mass spectrometric genetic analysis (“Asan-Panel”), interpretation of the results and drafting of the manuscript. WSK, JSL, SWK and DHL contributed to the interpretation of results and drafting of the manuscript. All authors read and approved the final manuscript.

## Pre-publication history

The pre-publication history for this paper can be accessed here:

http://www.biomedcentral.com/1471-2407/13/606/prepub
